# Fibrolamellar Hepatocellular Carcinoma (FLHCC) in a Young Patient Presenting With Nausea and Vomiting After a Greasy Meal

**DOI:** 10.7759/cureus.55863

**Published:** 2024-03-09

**Authors:** Mohamed Ismail, Sahiba Singh, Menna-Allah Elaskandrany, David s Kim, Yazan Abboud, Michael Bebawy, Abena Oduro, Ritik mahaveer Goyal, Omar Mohamed, Weizheng Wang

**Affiliations:** 1 Department of Medicine, Rutgers University New Jersey Medical School, Newark, USA; 2 College of Osteopathic Medicine, Michigan State University, East Lansing, USA; 3 Department of Internal Medicine, Lenox Hill Hospital, New York, USA; 4 Department of Internal Medicine, Rutgers University New Jersey Medical School, Newark, USA; 5 Department of Medicine, Saint Barnabas Medical Center, Livingston, USA; 6 Gastroenterology and Hepatology, Rutgers University New Jersey Medical School, Newark, USA

**Keywords:** non specific abdominal pain, elevated liver enzyme, nausea and vomiting, hepatic lesion, fibrolamellar hepatocellular carcinoma

## Abstract

Fibrolamellar hepatocellular carcinoma (FLHCC) is a rare and distinct subtype of liver cancer, predominantly affecting younger patients without underlying liver diseases. This case report discusses a unique presentation of FLHCC in a 38-year-old male with a past medical history of a well-controlled seizure disorder.

The patient presented with nausea, vomiting, and abdominal pain following a fatty meal. Laboratory tests revealed elevated liver enzymes and anemia, and imaging showed a large hepatic lesion. Initial management included a septic workup and broad-spectrum antibiotics. However, a liver biopsy performed subsequently revealed the presence of FLHCC. The patient underwent a successful open right hepatectomy and was managed postoperatively with consideration of his seizure disorder. Follow-up at six months showed a stable postoperative condition without any evidence of recurrence.

The diagnosis of FLHCC is challenging due to its rarity and nonspecific presentation. The case emphasizes the importance of considering FLHCC in the differential diagnosis of hepatic lesions, particularly in young patients. Surgical resection remains the primary treatment modality.

This case underscores the importance of a thorough evaluation of hepatic lesions, especially in younger patients. It also illustrates the complexities in managing patients with FLHCC, requiring a multidisciplinary approach for optimal outcomes. Further research is necessary to better understand the pathogenesis of FLHCC and to develop more effective treatment strategies.

## Introduction

FLHCC is a rare malignancy with a relatively unknown etiology and comprises about 1% of cases of liver neoplasms [1-4]. Patients diagnosed with hepatocellular carcinoma (HCC) generally belong to an older age group and often exhibit coexisting cirrhosis and elevated liver enzyme levels. In contrast, FLHCC predominantly affects a younger demographic, typically between the ages of 14 and 33. Notably, FLHCC occurs in the absence of cirrhosis and is usually not associated with any preexisting diseases [1-6].

Like HCC, FLHCC can invade hepatic veins and manifest as Budd-Chiari syndrome or cause Caval compression syndrome and obstructive jaundice by mass effect due to its large size [5]. Rarely, it can present as gynecomastia in males, fulminant liver failure, recurrent deep vein thrombosis, hypoglycemia, or encephalopathy [4,7]. Some studies have also demonstrated a female preponderance of FLHCC [3,7].

FLHCC does not arise from the common risk factors associated with HCC. Instead, it may be linked with various metabolic paraneoplastic disorders, such as abnormalities in vitamin B12-binding protein, sex steroid metabolism, ammonia processing, neurotensin synthesis, and gonadotropin production, as well as certain genetic syndromes like Gardner syndrome and Fanconi anemia [4,5]. The clinical picture is diverse, but all of these conditions need to be kept in mind as they can be underlying to this disease process.

Symptoms of FLHCC are nonspecific and can range anywhere from the patient being asymptomatic to having nausea, abdominal fullness, weight loss, hepatomegaly, and abdominal pain, which is the most common symptom [1,4,5]. About 50% of patients affected present at advanced stages when the carcinoma has already metastasized, negatively affecting their clinical course [1,6]. However, if detected early enough, surgical resection significantly improves survival outcomes [1,7,8]. The low incidence of the carcinoma makes it challenging to recruit patients for clinical trials, and as a result, treatment options for FLHCC are limited [9,10]. Our case presents another incidence of a novel malignancy, where nonspecific symptoms and unusual patient presentation posed a challenge for diagnosis.

## Case presentation

A 38-year-old male patient with a history of a well-controlled seizure disorder managed on 150 mg of lamotrigine daily presented to the hospital reporting nausea, vomiting, and mild, diffuse abdominal pain that commenced following the consumption of a fatty meal the night before. He recalled a similar episode of abdominal pain occurring four months prior, which had resolved spontaneously, leading him to forgo medical consultation. Upon physical examination, the patient exhibited normal vital signs, although tenderness was observed in the right upper quadrant. Laboratory tests revealed elevated levels of liver enzymes, including alanine aminotransferase (ALT), aspartate aminotransferase (AST), and alkaline phosphatase. Additionally, anemia was noted with a hemoglobin (HGB) level of 10.9, alongside increased white blood cell (WBC) counts and a heightened procalcitonin level (Table 1).

**Table 1:** Laboratory values on admission. WBC, white blood cell; AST, aspartate transaminase; ALT, alanine transaminase

Lab test	Patient values	Reference range
Hemoglobin (HGB)	10.9 g/dl	14-18 g/dl
WBC	12 K/µl	4-11 K/µl
Alkaline Phosphatase	153 U/L	40-130 U/L
Bilirubin Total	0.9 mg/dl	<= 1.0 mg/dl
AST	48 U/L	0-40 U/L
ALT	124 U/L	0-41 U/L
Procalcitonin	3.78 ng/ml	<0.50 ng/ml

**Figure 1:**
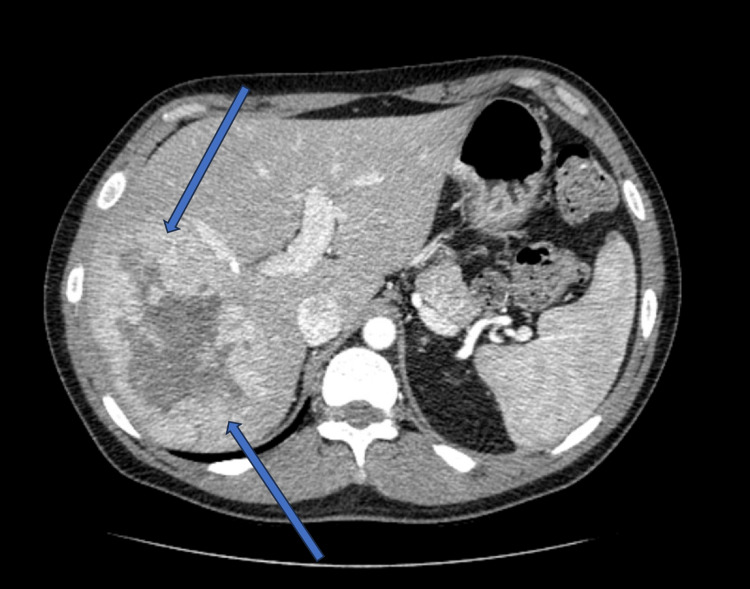
CT scan of the abdomen and pelvis with IV contrast A large hepatic lesion in the right lobe, measuring approximately 10.0 x 6.4 x 10.4 cm, with heterogeneous attenuation.

Tests for hepatitis B surface antigen (HBsAg) and hepatitis C virus antibody (HCV Ab) were negative. An abdominal ultrasound detected a 10.3 x 6.6 x 10.0 cm mass in the right hepatic lobe. Further assessment with computed tomography (CT) scan of the abdomen and pelvis with Intravenous (IV) contrast confirmed the presence of a large hepatic lesion in the right lobe, measuring approximately 10.0 x 6.4 x 10.4 cm, with heterogeneous attenuation (Figure 1).

Notably, tumor markers, including alpha-fetoprotein (AFP) and carcinoembryonic antigen (CEA), were within normal ranges. The initial clinical impression was a hepatic abscess, although a necrotic liver tumor was considered a differential diagnosis. Consequently, infectious disease specialists were involved, and the patient was subjected to a septic evaluation (including blood and urine cultures, and chest x-ray) and commenced on broad-spectrum antibiotics (piperacillin/tazobactam 3.375 g IV every 6 hours and metronidazole 500 mg every 8 hours).

**Figure 2:**
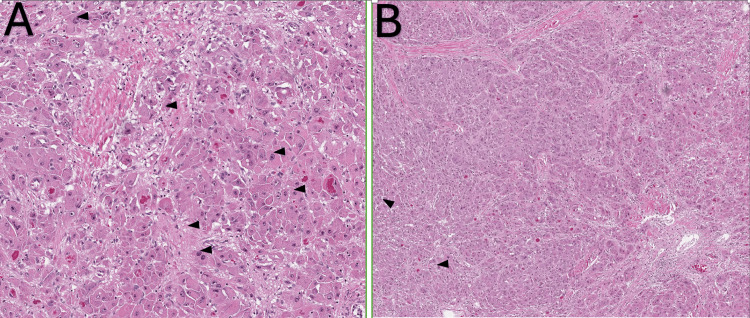
Microscopic Histological Patterns of FLHCC (A) Malignant cells, characterized by dyscohesive cells with enlarged nuclei, prominent nucleoli, oncocytic cytoplasm containing intra-cytoplasmic lumens and granules, with background fibrosis and granular debris (H&E, high power magnification). (B) Enlarged nuclei, oncocytic cytoplasm, with background fibrosis and granular debris (H&E, low power magnification).

An interventional radiology-guided liver biopsy was performed. Results from the septic workup were negative. Histopathological examination of the biopsy specimen revealed malignant cells, characterized by dyscohesive cells with enlarged nuclei, prominent nucleoli, oncocytic cytoplasm containing intra-cytoplasmic lumens and granules, and occasional fibrous tissue fragments and granular debris. These pathological findings were highly suggestive of fibrolamellar hepatocellular carcinoma (Figure 2).

**Figure 3:**
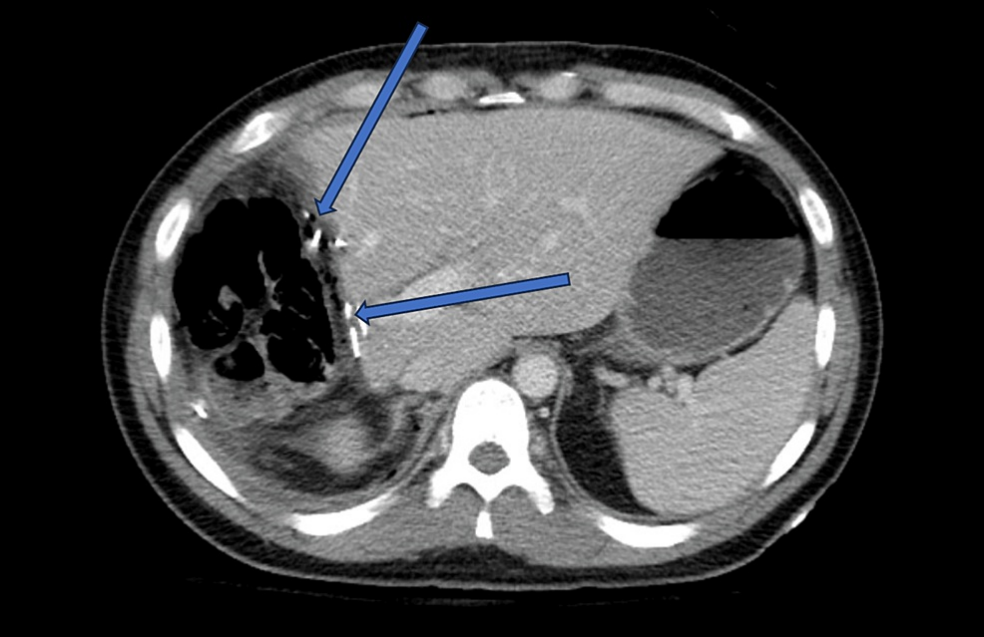
CT scan of abdomen and pelvis With IV Contrast Status post right hepatectomy for FLHCC

The antibiotic regimen was discontinued following this diagnosis, and the patient was referred to surgical oncology for further management. The investigation for metastasis using a whole-body CT scan yielded negative results, leading to the formulation of a surgical plan to resect the tumor. The patient underwent a successful open right hepatectomy, and a Jackson-Pratt (JP) drain was placed in the right upper quadrant during the procedure (Figure 3).

**Figure 4:**
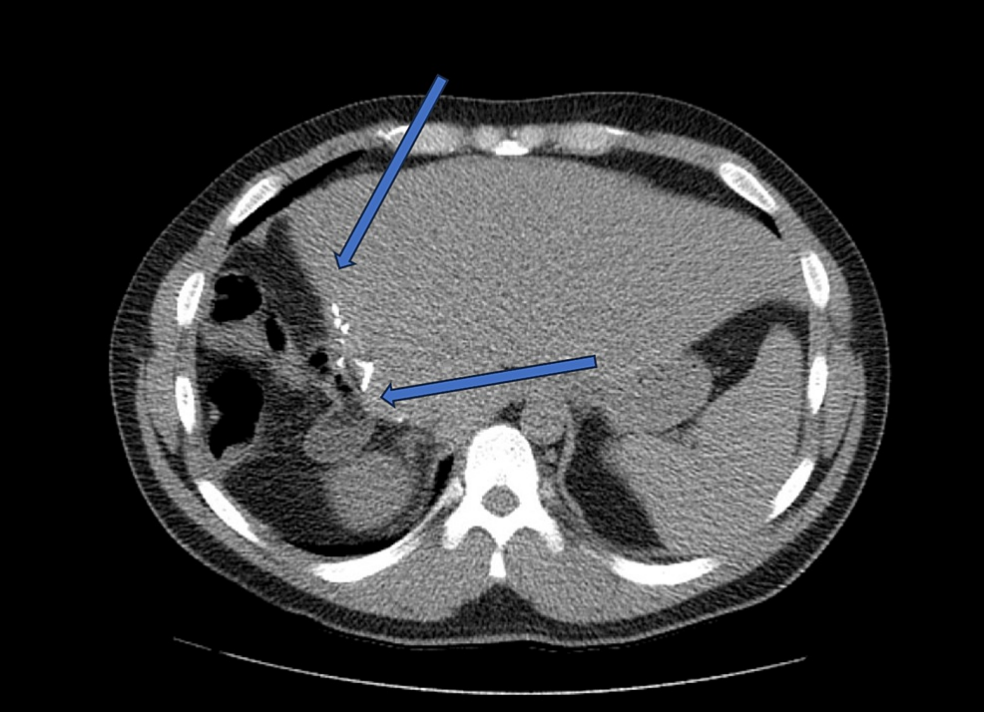
CT scan of abdomen and pelvis with IV contrast Six-month follow-up CT scan demonstrated a stable postoperative condition, with no enhanced hepatic lesions observed.

Postoperatively, the patient had an uneventful recovery in the post-anesthesia care unit (PACU) and was subsequently transferred to the general ward. The JP drain was removed, and following a neurology department evaluation for epilepsy management, the patient was discharged in stable condition. A six-month follow-up at the surgical oncology outpatient clinic revealed the patient to be asymptomatic, and a follow-up CT scan of the abdomen and pelvis with IV contrast showed a stable postoperative state, with no enhancing hepatic lesions noted (Figure 4).

## Discussion

The uncommon occurrence and indistinct clinical findings of FLHCC can lead to a failure in early detection [3,4,7]. This emphasizes the diagnostic challenges in managing this carcinoma, and as a result, many patients present at an advanced stage in the disease [1,6].

Diagnosis of FLHCC is usually achieved by the patient's clinical presentation and imaging [10]. Ultrasonography is commonly used for evaluation; however, features are usually nonspecific and necessitate further imaging by either CT or magnetic resonance imaging (MRI) [1,4,11]. Lesions are typically hypervascular, solitary, well-delineated, and may have a central scar [1,4,11]. Indeterminate lesions by imaging may need to undergo fine needle aspiration or biopsy for definitive diagnosis [4]. FLHCC can be confused for hypervascular liver lesions such as hepatic adenomas, focal nodular hyperplasia, hemangiomas, and hepatocellular carcinoma and must be differentiated [4,11].

Furthermore, nearly all cases of FLHCC have been identified with the presence of an oncogenic DNAJB1-PRKACA fusion protein and a heightened capacity for serum vitamin B12 binding, both of which were not evaluated in our case [2-6,10]. Although they are not specific for this carcinoma, these findings, along with clinical presentation and imaging, can help provide a diagnosis. The DNAJB1-PRKACA fusion gene can also aid in differentiating FLHCC from HCC since it is not present in the latter case [8].

Pathology remains the gold standard for diagnosis, and FLHCC tumor cells will demonstrate an eosinophilic cytoplasm with prominent nuclei and a fibrous stroma arranged in parallel lamellae [5,8,10].

In scenarios where hepatocellular carcinoma (HCC) is suspected in patients with non-cirrhotic livers and diagnostic imaging fails to conclusively identify HCC-as exemplified by our patient with a non-cirrhotic, healthy liver where imaging did not confirm liver cancer, yet the differential diagnosis raised the possibility of either liver cancer or a hepatic abscess-a histological examination through liver biopsy may become imperative [12]. The clinical team, assessing the necessity of a fine needle biopsy, engaged in thorough discussions with the patient regarding the risks and benefits of the procedure. Given the relatively low risk of tumor seeding, as documented in prior studies, the patient consented to the biopsy.

The practice of utilizing image-guided liver biopsies, conducted via CT scans and ultrasound, is affirmed as the premier diagnostic standard for lesions that are indeterminate upon imaging [13]. Pain, ranging from mild discomfort to more pronounced discomfort, emerges as the most prevalent complication, affecting up to 84% of patients who undergo liver biopsy. While serious adverse events such as gallbladder perforation, bile peritonitis, haemobilia, pneumothorax, or hemothorax are exceedingly rare, they highlight the inherent risks of the procedure [12]. Notably, despite the predominance of arteriolar severe hemorrhages, the application of ultrasound guidance has been shown to not significantly mitigate bleeding risks, though it effectively reduces the frequency of overall complications [12].

The potential for needle biopsies to disseminate cancer cells along the tract of needle insertion has been a subject of concern, although the precise dynamics and actual risk remain partially understood [12]. A widely referenced meta-analysis reported a 2.7% incidence of cancer cell dissemination in 1340 biopsies. However, incorporating findings from three additional, more contemporary studies suggests the possibility of significantly lowering these dissemination rates to below 1% [12,14,15]. Furthermore, the clinical significance of seeding has generally been minimal, with most instances being effectively managed through surgical or ablative interventions, thereby not adversely impacting patient morbidity or mortality [12].

Surgical resection remains the mainstay for treatment, although these tumors are aggressive, and recurrence of the carcinoma is common because of the large tumor size and the majority of patients presenting at an advanced stage [1,3,4,8,10]. Patients unable to undergo resection due to metastasis or major vessel involvement have a 5-year survival rate of 0-5% in comparison to a 58-82% rate of those who underwent surgery [4,7,8]. Favorable prognostic indicators include female gender and locoregional disease without lymph node involvement [3,4,10].

Chemotherapy can be used as a treatment option before or after surgery for FLHCC. However, there is limited research on the most effective chemotherapeutic regimen due to the low occurrence of this type of cancer. It should be noted that there are currently no reported neoadjuvant/adjuvant systemic therapies that have improved survival rates for patients with resectable FLHCC [1,16]. 

New research has focused on using the latest understanding of FLHCC's pathogenesis and molecular genetics to improve treatment options, along with systemic chemotherapy. For example, a current multi-institutional, randomized controlled trial is exploring the effectiveness of combining mTOR inhibition with estrogen suppression to treat FLHCC. Additionally, studies have shown that FLHCC expresses increased levels of epithelial growth factor receptor and transforming growth factor beta, making these potential targets for future treatments [16].

## Conclusions

FLHCC represents a significant diagnostic and therapeutic challenge due to its rarity, unique demographic profile, and often indistinct clinical presentations. This case underscores the importance of maintaining a high index of suspicion for FLHCC in young patients presenting with hepatic lesions, even in the absence of traditional risk factors for liver neoplasms. The fact that FLHCC typically presents at a more advanced stage and the lack of specific systemic therapies highlight the urgent need for early detection and the development of effective treatment strategies.

The emerging understanding of the molecular pathogenesis of FLHCC, including the DNAJB1-PRKACA gene fusion and the role of increased serum vitamin B12 binding capacity, offers new avenues for research and potentially targeted therapies. Continued research is critical to unravel the complexities of FLHCC, improve diagnostic methods, and develop more effective treatment modalities, thereby improving outcomes for this unique and challenging patient population.
